# What Should Guide the Performance of Venous Resection During Pancreaticoduodenectomy for Pancreatic Ductal Adenocarcinoma with Venous Contact?

**DOI:** 10.1245/s10434-020-09568-2

**Published:** 2021-01-21

**Authors:** Julie Navez, Christelle Bouchart, Diane Lorenzo, Maria Antonietta Bali, Jean Closset, Jean-Luc van Laethem

**Affiliations:** 1 Medico-Surgical Department of Gastroenterology, Hepatopancreatology and Digestive Oncology, Erasme Hospital, Université Libre de Bruxelles, Brussels, Belgium; 2 Department of Radiotherapy, Institut Jules Bordet, Brussels, Belgium; 3 Department of Radiology, Institut Jules Bordet, Brussels, Belgium

## Abstract

Complete surgical resection, most often associated with perioperative chemotherapy, is the only way to offer a chance of cure for patients with pancreatic cancer. One of the most important factors in determining survival outcome that can be influenced by the surgeon is the R0 resection. However, the proximity of mesenteric vessels in cephalic pancreatic tumors, especially the mesenterico-portal venous axis, results in an increased risk of vein involvement and/or the presence of malignant cells in the venous bed margin. A concomitant venous resection can be performed to decrease the risk of a positive margin. Given the additional technical difficulty that this implies, many surgeons seek a path between the tumor and the vein, hoping for the absence of tumor infiltration into the perivascular tissue on pathologic analysis, particularly in cases with administration of neoadjuvant therapy. The definition of optimal surgical margin remains a subject of debate, but at least 1 mm is an independent predictor of survival after pancreatic cancer surgical resection. Although preoperative radiologic assessment is essential for accurate planning of a pancreatic resection, intraoperative decision-making with regard to resection of the mesenterico-portal vein in tumors with a venous contact remains unclear and variable. Although venous histologic involvement and perivascular infiltration are not accurately predictable preoperatively, clinicians must examine the existing criteria and normograms to guide their surgical management according to the integration of new imaging techniques, preoperative chemotherapy use, tumor biology and molecular histopathology, and surgical techniques.

Pancreatic ductal adenocarcinoma (PDAC) is an aggressive and devastating cancer, with a 5-year overall survival (OS) of approximately 8.5% for all stages combined.^1^^,^^2^ Unfortunately, its incidence is rising, and PDAC soon will become the second most common cause of death by cancer worldwide. Although surgical resection has always been considered the only potentially curative treatment for non-metastatic PDAC, the recent development of active chemotherapies and modern radiotherapy techniques has led to increasing consideration of these options in combination with surgery to improve survival and chances of remission.

Resectability of PDAC depends on its contacts with surrounding vascular structures. In the past, arterial and/or venous infiltration was considered a contraindication for curative surgery due to the difficulty of vascular dissection and the associated poor prognosis. Currently, pancreatectomy combined with arterial resection still is not recommended (except in selected cases), but venous resection and reconstruction (VRR) are routinely performed with acceptable postoperative and oncologic outcomes.^3^^–^5 In this setting, neoadjuvant therapy (NAT), including chemotherapy and radiotherapy, may influence the disease course, as shown in two recently published randomized controlled trials.^6^^–^8

The main goal of surgery for PDAC is a complete oncologic resection with free surgical margins (R0 resection). This is the most important prognostic factor that can be influenced by the surgeon.^9^ The anatomic location of the pancreatic head and uncinate process, surrounded by superior mesenteric vessels, coeliac artery, and collaterals, makes the surgical dissection very delicate, with many cancers removed, leaving remnant tumoral cells on the resection margin, or by cutting very close to it, exposing the patient to early cancer recurrence.^10^^,^^11^

To decrease the risk of positive margins in cases of tumoral vascular contact or involvement, which is difficult to detect intraoperatively on a macroscopic scale, resection of the mesenteric and/or portal vein (SMV/PV) may be useful. However, given the additional technical difficulty of simultaneous VRR during pancreatectomy, many surgeons persist in trying to separate the tumor from the vein, with the risk of venous injury and uncontrolled hemorrhage. They dissect with the hope that the venous wall, perivascular tissue, or both are shown by pathologic analysis not to be invaded due to the efficacy of NAT. Despite good intentions for entire removal of the tumor, the surgeon frequently is disappointed when the pathologic report shows R1 resection with a positive margin or margins.

Whereas the need for R0 resection is clearly established in consensus guidelines for PDAC, intraoperative surgical decision-making regarding the resection of the SMV/PV in tumors with venous contact and/or involvement to obtain negative margins remains unclear, and is even more unclear in the emerging setting of NAT. This review aimed to critically analyze the existing evidence-based literature to identify factors that may help in this decision-making process.

## Definition of PDAC Resectability: Radiologic Staging as the Key for Decision-Making

The criteria for non-metastatic PDAC resectability, established to guide treatment strategy according to locoregional extension, are based on radiologic findings, resulting in three sub-entities: resectable, borderline resectable, and locally advanced PDAC. Accurate staging is essential for selection of patients for NAT or upfront surgery but is sometimes challenging, especially with regard to tumor-vascular contacts.

A multi-phase contrast-enhanced computed tomography (CT) currently is the best-validated method for PDAC staging.^12^ In each vascular phase, the length and circumference of tumor-vessel contacts, the presence of vascular infiltration, contour abnormalities, and thrombosis should be carefully assessed. However, the accuracy of CT for assessment of vascular invasion is not very high, with a sensitivity of 63% and a specificity of 92%.^13^ Some factors can interfere with radiologic interpretation, including imaging resolution, quality of injection phases, presence of pancreatitis, and artifacts secondary to biliary prosthesis.

Several groups have formulated criteria (Table 1), focused on the tumor-vessel interface.^14^^–^19 The main problem with interpretation of these criteria is the terminology used by each group because it is potentially subjective and does not necessarily have the same significance across groups. For example, terms such as “contact,” “abutment,” “involvement,” “impingement,” “narrowing,” and “encasement” can vary in intended meaning and extent.

**Table 1:** Criteria defining borderline resectable pancreatic adenocarcinoma

	SMV/PV	SMA	CHA	CA
MDACC–2006^15^	Short-segment occlusion, suitable for reconstruction	Abutment ≤180°	Short-segment abutment	··
AHPBA–2009^14^	Abutment or encasement, with or without short-segment occlusion, suitable for reconstruction	Abutment ≤180°	Short-segment encasement or direct abutment	··
Alliance–2013^16^	Tumor/vessel interface ≥180° and/or reconstructable occlusion	Tumor/vessel interface <180°	Reconstructable, short-segment tumor/vessel interface	Tumor/vessel interface <180°
Japan Pancreas Society–2016^17^	Abutment / encasement ≥180° or occlusion	Abutment/encasement <180°, without contour irregularity	Abutment/encasement without contour irregularity of PHA and/or CA	Abutment/encasement <180°, without contour irregularity
NCCN–2017^18^	Contact >180° or ≤180° with contour irregularity or thrombosis, but suitable for reconstruction	Contact ≤180°	Contact with CHA without extension to CA or hepatic bifurcation, suitable for reconstruction	Head: no extension Body/tail: contact ≤180° or contact >180° without aorta or GDA involvement
IAP International Consensus–2018^19^^a^	Contact ≥180° or bilateral narrowing/occlusion	Contact <180° without deformity/stenosis	Contact without contact with the PHA and/or CA	Contact <180° without deformity/stenosis

SMV/PV, superior mesenteric vein/portal vein; SMA, superior mesenteric artery; CHA, common hepatic artery; CA, celiac artery

MDACC, MD Anderson Cancer Center; AHPBA, Americas Hepato-Pancreato-Biliary Association; NCCN, National Comprehensive Cancer Network; GDA, gastroduodenal artery; PHA, proper hepatic artery; IAP

^a^Includes also biologic (CA 19-9 levels) and clinical aspects

Overall, PDAC is considered to be resectable in the absence of venous involvement or any abutment with arterial vasculature. Major controversies pertain to the precise definition of “borderline resectable,” especially with regard to the SMV/PV (Table 1). The MD Anderson Cancer Center includes any short-segment venous occlusion suitable for reconstruction,^15^ whereas the Americas Hepatopancreatobiliary Association considers all abutment, encasement, or short-segment occlusion as venous involvement,^14^ and the National Comprehensive Cancer Network (NCCN) requires an SMV/PV contact exceeding 180°, venous irregularity, or thrombosis suitable for reconstruction (similar to the Alliance Group and Japan Pancreas Society).^17^^,^^18^ These variations highlight the ambiguity involved in evaluating and comparing outcomes from different studies worldwide.

Special attention should be paid in cases of SMV/PV contact less than 180°, considered to be resectable in most definitions. Indeed, a tumor–vessel interface of less than a hemi-circumference does not mean that the vein or the perivenous tissue is not invaded and does not need to be resected. Some classifications and normograms have been established to assess the vascular involvement and predict the vein invasion with relatively good sensitivity and accuracy, but these cannot identify the nature of perivenous tissue.^20^^,^^21^ Histologic vein invasion is nevertheless observed in 20% of patients with tumoral contact of 180° or less or no tumoral contact.^21^ Therefore we need more reliable criteria in the future to guide surgical management and performance of complete oncologic resection.

**Fig. 1.**
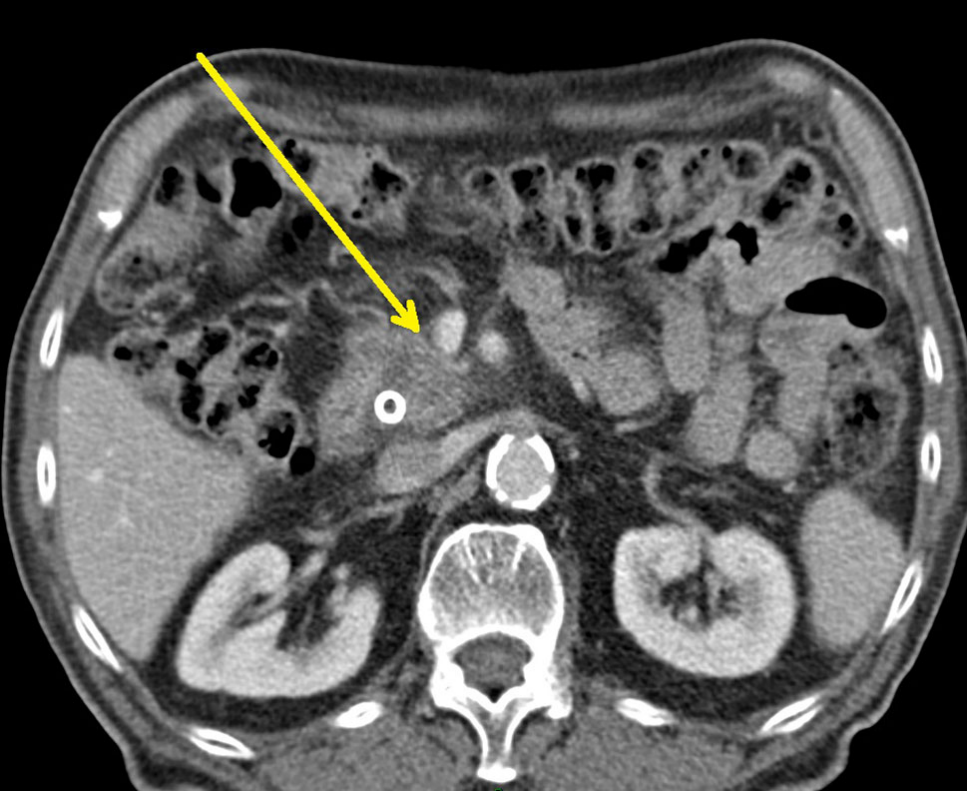
Pancreatic tumor with a PV/SMV contact smaller than 180° without deformity (arrow) in a patient undergoing pancreaticoduodenectomy without vein resection after neoadjuvant treatment. The final pathology showed the presence of malignant cells at the SMV/PV margin (R1 resection). SMV, superior mesenteric vein; PV, portal vein

Additional difficulties exist for radiologists, including imaging changes after NAT and detection of micrometastasis. First, preoperative CT restaging after NAT may show poor specificity for differentiation of residual viable tumor and posttreatment-induced changes (with no viable tumor) at the tumor–vessel interface due to a lack of contrast resolution.^22^ Pancreatic ductal adenocarcinoma is composed of fibrous and dense stroma, and after chemoradiation, cancer cells may decrease or disappear, leaving in their place a fibrotic and necrotic tissue that cannot be differentiated from residual cancer (Fig. 1). Moreover, radiation therapy may induce locoregional edema, and bile duct endoprosthesis placement may bring on inflammatory alterations.

Second, intraoperative detection of liver or peritoneal micrometastasis is the main cause of aborted surgery despite resectable disease at preoperative CT.^23^ Missed hepatic lesions frequently are subcapsular and infra-centimetric, which makes diagnosis almost impossible by low-resolution CT.

During the last decade, improvements in imaging tools and techniques have strengthened the crucial role of the radiologist in PDAC management. Development of high-resolution imaging with thin-slice thickness and multi-planar reconstruction allows better analysis of tumoral extension and the tumor–vessel interface. Magnetic resonance imaging (MRI) performed with specific sequences appears to be very promising, especially in combination with metabolic imaging and conventional CT.^24^^,^^25^ Diffusion-weighted MRI can identify responding patients upon PDAC restaging after NAT thanks to apparent diffusion coefficient-mapping by monitoring of treatment-induced changes in PDAC and also is more sensitive than CT for imaging of small PDAC liver metastases (83% vs 45%).^23^^,^^25^ In our opinion, MRI should be prospectively incorporated as part of PDAC staging and restaging after NAT, especially for vascular contact (re)assessment and tumoral changes.

## The Role of NAT is Emerging But Still Under Exploration

The role of NAT in (borderline) resectable PDAC is not clearly established due to a low level of proof, but nevertheless is increasingly used and even recommended for borderline and high-risk tumors (with locoregional lymph nodes or high CA 19.9 levels). Contemporary chemotherapeutic combination therapies, particularly FOLFIRINOX and gemcitabine/nab-paclitaxel, have demonstrated better efficacy than single agents in the metastatic setting.^26^^,^^27^ Growing interest is focused on using these combinations as NAT, which currently is under evaluation.^28^^,^^29^ According to the recent Dutch phase 3 PREOPANC-1 trial evaluating the benefit of preoperative chemoradiotherapy (gemcitabine-based, 36 Gy in 15 fractions) compared with immediate surgery, NAT was associated with a higher rate of R0 resection (71% vs 40%, *p* < 0.001) and better disease-free survival (DFS) (16 vs 14.3 months; *p* = 0.096), but no statistically significant benefit in terms of OS by intention-to-treat was demonstrated.^8^

At American Society of Clinical Oncology (ASCO) 2020, the international phase 2 ESPAC-5F trial presented preliminary results, randomly assigning 90 patients with borderline PDAC to receive NAT (GEMCAP, FOLFIRINOX, or chemoradiotherapy) or immediate surgery. No significant differences in R0 resection rates were observed, but administration of NAT showed a survival benefit at 1 year. In Japan, gemcitabine combined with S-1 in the neoadjuvant setting showed promising results in a randomized phase 2/3 trial, with a better survival than upfront surgery, although confirmation of the results are awaited.^30^

Recently, a randomised phase 2 study explored preoperative therapy with FOLFIRINOX versus gemcitabine/nab-paclitaxel, and although a bit disappointing in terms of DFS impact, both combinations were shown to provide a significant pathologic major response and limited toxicity, which are highly desirable in this difficult setting.^31^ Currently, a modified version of FOLFIRINOX (mFOLFIRINOX), by eliminating the bolus of fluorouracil and lowering the dose of irinotecan or by a starting dose 80% the intensity of FOLFIRINOX, is frequently used to improve its tolerability without a negative impact on tumor response.^32^ Which optimal regimen and timing of NAT should be administered currently remains an open question and requires further trials.

Questions also remain regarding the impact of NAT on venous invasion. Biologically, PDAC is an extremely infiltrative neoplasm. The oncologic reason for SMV/PV tumoral involvement remains controversial and has been explained as either the reflection of aggressive tumor biology or a consequence of tumor size and/or the anatomic proximity of the pancreatic head and uncinate process to mesenteric vessels.^33^^,^^34^ The heterogeneous interindividual response to chemotherapy and the high rate of tumor recurrence despite a complete surgical resection argues for biomolecular mechanisms over tumor topography. Delpero et al.^35^ observed that resected PDACs requiring VRR were more aggressive tumors, with histologic factors of poor prognosis such as poor differentiation, highlighting the need for NAT to downsize the tumor.

On the other hand, as a strong argument for the topographic hypothesis, complete tumor removal is one of the most important prognostic factors for long-term survival.^36^ Interestingly, Mierke et al.^37^ analyzed recurrence patterns after PDAC resection with VRR. They observed that despite en bloc vascular resection, the true pathologic invasion of SMV/PV constitutes an independent risk factor for OS and DFS, noting a higher incidence of liver metastasis compared with the absence of SMV/PV infiltration. They hypothesized a dissemination of malignant cells through the portal system once the tumor invades SMV/PV to finally reach the liver.

Currently, the biologic and molecular effects of NAT on PDAC and venous tumoral contact are unknown. By targeting the tumor both locally and systemically, NAT may have an impact on histopathologic features of pancreatic specimens including tumor margin, lymph node positivity, and vascular, perineural, lymphatic, and peripancreatic adipose tissue invasion.^8^^,^^38^^–^42 It also targets and reduces micrometastatic spreading before and during surgery.^43^ A recent meta-analysis showed that patients who underwent NAT had a twofold greater probability of negative lymph node status than patients undergoing upfront surgery, with lower rates of perineural/lymphatic invasion and R1 resection.^38^

Frequently, NAT induces extensive fibrosis in the tumor, pancreatic parenchyma, and peripancreatic tissue. The expected effects are shortening of the tumor–vessel interface and destruction of isolated cancer cells that tend to spread around the tumor. The histopathologic response after NAT has been graded according to different classifications. The most currently used classification is the College of American Pathologists (CAP) tumor regression grading system.^44^ Findings show that patients with a complete pathologic response (CAP 0) or minimal residual tumor (CAP 1) have a longer survival than patients with higher grades (mean OS and DFS of 54 and 44 months for CAP 0 or 1 vs 44 and 28 months for CAP 2, respectively).^45^ The rates range from 6 to 10% for CAP 0 and from 13 to 28% for CAP 1 after NAT including chemoradiotherapy and/or chemotherapy alone.^41^^,^^45^^–^48 It should be emphasized that no proven NAT regimen has been shown to date, and additional trials should be conducted in this setting.

Because tumoral reduction and histologic changes after NAT cannot be precisely quantified by preoperative imaging or even intraoperatively when residual fibrosis and tumoral tissue are not distinguishable, VRR plays an important role in the removal of any residual cells around and inside the SMV/PV that have not completely responded to NAT administrated for PDAC with venous contact. Biologic and molecular analysis of this vascular margin could help us better understand the behavior of PDAC around the SMV/PV and the role of NAT in this setting. To evaluate the degree of tumor response to NAT preoperatively, the development of novel imaging techniques such as MRI-specific sequences and metabolic imaging could be very helpful in answering the question of when the vein should be resected.^24^^,^^25^

## Definition of a “TRUE” R0 Resection

The primary goal of a curative PDAC resection is to perform an R0 resection with margins free of tumor cells.^10^ Eight different margins are identified on a specimen of pancreaticoduodenectomy, from transection, dissection, or free surfaces. These include pancreatic and bile duct margins, proximal gastric/duodenal and distal duodenal/jejunal margins, anterior and posterior surface margins, a superior mesenteric artery (SMA) margin (or uncinate margin), and a SMV/PV margin (or mesenterico-portal vein groove). The definition of the margin size for PDAC surgery remains a matter of debate and is not equivalent worldwide.

Before 2010, R0 resection was considered when no microscopic evidence of tumor was observed at any of the specimen’s cutting edges.^49^ However, given the aggressive behavior of PDAC and its invasive growth pattern, some authors have questioned the value of this margin, judging it inappropriate for describing a true R0 resection and stating that a larger margin is needed.^10^^,^^50^ Whereas a few studies did not report significant differences in oncologic outcomes between an R0 greater than 0 mm and an R0 greater than 1 mm, others have suggested that extending the cutoff to 1, 1.5, or even 2 mm could significantly improve survival.^10^^,^^51^

The UK Royal College of Pathologists reappraised the definition of R0 resection, requiring at least 1 mm of free margin, a guidance recommended mainly in Europe and by expert groups, but not in many centers worldwide, which still use the 0 mm clearance, as defined by the Union for International Cancer Control (UICC).^11^ This discrepancy makes meta-analysis and study comparison difficult, with R0 rates varying from 29 to 71% and with associated survival differing by 1 year (median survival range, 14–35 months).^52^^,^^53^

Additionally, due to the absence of consensus related to margin size, the wide variation between R0 resection rates also is due to the lack of standard protocols for specimen examination. Dissection of a pancreatoduodenectomy specimen is complex, and different approaches exist that may influence assessment of surgical margins. This represents a collaborative process in the operating room between surgeons and pathologists to mark the specimen with multicolor ink and to stain the specimen adequately for reliable identification of each margin.^54^

The gross specimen dissection technique can be performed in different ways.^50^ Traditionally, the specimen is opened longitudinally and sliced along a plane defined by the main pancreatic and common bile ducts. Further dissection by slicing in another plane is required, which makes tumor orientation and spatial representation much more difficult for the pathologist.

A more recent method consists of axial slicing in the craniocaudal dimension perpendicular to the longitudinal axis of the second duodenum and description by quadrants in anteroposterior and mediolateral dimensions. Therefore, the entire pancreatic surface on every slice can be examined readily for a more reliable determination of all surgical margins, including the SMA and SMV/PV margins.^50^

Although no international consensus exists regarding the R0 margin definition or standard gross dissection protocol for pancreatic specimens, the 1-mm rule is increasingly accepted in European centers using the axial slicing method. These measures expose the patient to a higher rate of R1 resection, but they reflect the reality of the disease and should guide our practice.

## Intraoperative Assessment of R0 Resection is Highly Challenging

No 100% reliable method exists for preoperative identification of SMV/PV tumoral invasion. In approximately 40% of patients undergoing VRR for suspicion of venous infiltration, only inflammatory adherences and fibrosis are observed at the final pathology secondary to NAT or local inflammatory response.^55^^,^^56^ Intraoperative frozen section is commonly used to ensure negative final margins after parenchymal pancreatic transection with an accuracy of about 90%.^57^ However, the most common cause of R1 resection during pancreatectomy is not related to the transection margin, which can lead to additional transection but is due to positive SMA and SMV/PV margins. These latter margins are less likely to be extended to further resection, especially when the dissection has been performed optimally.^54^ To our knowledge, no study has evaluated the accuracy of frozen sectioning on these margins because this probably is difficult to assess for a number of reasons. First, tumors with vascular involvement are frequently treated with NAT, resulting in frozen sections that are more complex to interpret due to a paucity of tumor cells, fibrosis, and inflammation. In this situation, immunohistochemistry may be the only tool helpful for diagnosis.

Second, to obtain a reliable diagnosis of margins, the dissection procedure must be carefully performed by serial slicing in a specific plane, as described earlier.^50^ Collecting a frozen section sample on a vascular margin before final dissection could alter the quality, precision, and accuracy of the definitive histopathologic analysis.

Finally, performing an additional VRR without en bloc resection exposes the surgeon to the possibility of tumor-splitting in cases of true vascular involvement and a non-reliable localization of the tumor–vessel interface because the specimen is in multiple pieces.

Intraoperative ultrasonography (IOUS) for better determination of the tumor-vessel interface can be helpful, even after NAT. A recent Dutch multicenter study found that IOUS changed the PDAC resectability status for 32% of patients who received neoadjuvant FOLFIRINOX.^58^ This promising diagnostic tool needs further evaluation for it to be a validated option in the management of PDAC.

## Outcomes After Pancreatectomy With Venous Resection

Whereas the use of simultaneous arterial resection during pancreatectomy is no longer recommended except in selected situations, the role of VRR is much more established and frequently performed in high-volume centers in the presence of limited lateral or circumferential involvement without venous occlusion. The impact of venous reconstruction on postoperative outcome is widely debated. Many authors have reported postoperative morbidity and mortality comparable with those of standard pancreatectomy, as shown in meta-analyses, national surveys, and large single-center studies (Table 2).^3^^,^^35^^,^^59^^–^63

**Table 2:** Meta-analysis of pancreaticoduodenectomy with venous resection

Authors	Studies (period)	Patients (PD+VR/PD)	Morbidity	Mortality	Resection rate	Histologic vein invasion (%)	3-Year OS	5-Year OS
Fancellu et al.^65^	23 (1998–2019)	1729/4308	OR 1.07; *p* = 0.65 (4% vs 4%)	OR 1.93; *p* = 0.002 (3% vs 2%)	R0: OR 0.60; *p* = 0.0001 (58% vs 68%)	65.8	OR 0.72; *p* = 0.0006 (20% vs 20%)	OR 0.57; *p* = 0.003 (10% vs 20%)
Peng et al.^67^	30 (1996–2017)	2186/9845	–	OR 1.71; *p* = 0.01 (5.2% vs 2.9%)	R0: OR 0.64; *p* < 0.001 (64.0% vs 71.3%)	–	–	–
Bell et al.^66^	16 (1996–2015)	1207/2938	OR 1.21; *p* = 0.14 (40% vs 36%)	OR 1.72; *p* = 0.04 (6% vs 4%)	R1: OR 1.59; *p* < 0.0001 (37% vs 32%)	64	OR 0.74; *p* = 0.24 (16% vs 19%)	OR 0.20; *p* = 0.002 (3% vs 8%)
Giovinazzo et al.^64^	27 (1996–2014)	1587/7418	OR 1.34; *p* = 0.03 (38.5% vs 32.1%)	RD 0.01; *p* = 0.02 (3.9% vs 3.0%)	R1: RD 0.09; *p* < 0.001 (37.0% vs 31.0%)	61	HR 1.48; *p* = 0.0004	HR 3.18; *p* > 0.001
Yu et al.^3^	22 (1994–2013)	794/2096	OR 1.01; *p* = 0.93 (38.4% vs 37.2%)	OR 1.49; *p* = 0.07 (5.5% vs 4.3%)	R0: OR 0.60; *p* < 0.001 (68.0% vs 76.0%)	61	OR 0.78; *p* = 0.20 (13.7% vs 19.2%)	OR 0.69; *p* = 0.03 (10.1% vs 12.5%)
Zhou et al.^59^	19 (1994–2011)	661/1586	OR 0.95; *p* = 0.67 (41.9% vs 44.0%)	OR 1.19; *p* = 0.48 (3.3% vs 3.7%)	/	56.9	OR 0.71; *p* = 0.09 (19.4% vs 26.6%)	OR 0.57; *p* = 0.06 (12.3% vs 17.0%)

PD, pancreaticoduodectomy; VR, venous resection; OR, odds ratio; RD; HR, hazard ratio

Nevertheless, other meta-analyses have observed an increased but small risk of higher postoperative mortality rates after VRR (3–6% vs 2–4%; *p* < 0.05), including more reoperations and postoperative bleeding, but these rates remain relatively low.^64^^–^67 Notably, all these analyses had some degree of bias, including the absence of randomized studies and differences in the administration of NAT. This latter point is important because currently, an increasing number of patients are receiving NAT, resulting in a harder and less trophic pancreas secondary to chronic obstruction of the pancreatic duct during the NAT period, making the pancreas at lower risk for pancreatic fistula.^3^^,^^54^^,^^66^ Hank et al.^68^ reported a 3.6-fold lower rate of clinically relevant postoperative pancreatic fistula for patients receiving NAT versus upfront resection for PDAC.

Does synchronous vein resection improve long-term survival? The oncologic value is not well-known given its technical complexity associated with the aggressive natural history of PDAC, the more advanced stage of the disease in cases of venous involvement, and the dismal prognosis. In many studies, the rate of R1 resection is indeed higher after pancreatectomy with VRR than after standard pancreatectomy, whereas the median survival is decreased (Table 3).^35^^,^^55^^,^^62^^,^^63^^,^^69^^–^87

**Table 3:** Studies since 2010 comparing R0 resection rate and survival after pancreaticoduodenectomy with versus without venous resection for pancreatic cancer (at least 40 venous resections).

Authors	Surgery	Sample (*n*)	R0 margin definition (mm)	R0 rate (%)	R0 SMV margin (%)	R0 SMA margin (%)	Median survival (months)	Vein invasion (%)
Han et al.^69^	PD+VR	106	0	78.4^a^	–	–	–	75.5
	PD	451		87.6				
Xie et al.^70^	PD+VR	138	Unknown	96.4	–	–	25.1^a^	92.0
	PD	239		94.1			29.3	
Mohammed et al.^71^	PD+VR	42	>1	69.0	–	–	17^a^	57.8
	PD	93		80.7			31.3	
Klein et al.^72^	PD+VR	40	Unknown	60.0	–	–	10.4	–
	PD	120		61.7			18.6	
Malleo et al.^62^	PD+VR	81	≥1	45.7^a^	SMV+SMA: (65.4)^a^		28	69.1
	PD	570		61.9	SMV+SMA: (77.9)		26	
Kleive et al.^73^	PD+VR	79	0/≥1^b^	22.0	–	–	21.1	–
	PD	208		45.0			17.1	
Addeo et al.^74^	PD+VR	91	≥1	43.0^a^	(55)	(35)	22	74.0
	PD	90		64.5	–	–	27	
Roch et al.^55^	PD+VR	90	Unknown	73.3	–	–	14	57.8
	PD	477		80.9			21	
Michalski et al.^75^	PD+VR	54	Unknown	44.4^a^	–	–	15.8	50.0
	PD	102		30.4			22.7	
Delpero et al.^35^	PD+VR	402	0	62.0^a^	–	–	21^a^	55.6
	PD	997		81.5			29	
Kulemann et al.^76^	PD+VR	131	Unknown	64.6^a^	–	–	21.6	–
	PD	208		76.2			19.7	
Murakami et al.^63^	PD+VR	435	0	69.7^a^	–	–	18.5^a^	59.5
	PD	502		77.7			25.8	
Wang et al.^77^	PD+VR	42	Unknown	81.0	–	–	20.0	100
	PD	166		78.3			26.0	
Jeong et al.^78^	PD+VR	46	Unknown	65.2^a^	–	–	16	65.2
	PD	230		85.2			12	
Hirono et al.^79^	PD+VR	99	0	70.7	–	–	16.6	57.6
	PD	206		78.6			21.3	
Wang et al.^80^	PD+VR	64	>1	18.8^a^	–	–	18^a^	75.8
	PD	58		55.2			31	
Ravikumar et al.^81^	PD+VR	230	≥1	37.1^a^	(63.9)^a^	(89.2)	18.2	–
	PD	840		48.4	(88.5)	(92.1)	18	
Kelly et al.^82^	PD	70	≥1	68.5	–	–	12.4^a^	–
	PD	422		74.9			19.3	
Gong et al.^83^	PD+VR	119	Unknown	100	–	–	13.3	95.8
	PD	447		100			20	
Banz et al.^84^	P+VR	51	≥1	49.0^a^	–	(42.3)	14.5	49.0
	PD	275		63.3		(37.6)	14.8	
Murakami et al.^85^	PD+VR	61	0	50.8^a^	–	–	14.7^a^	63.9
	PD	64		71.9			26.7	
Turley et al.^86^	PD+VR	42	Unknown	73.8	–	–	21.1	–
	PD	162		72.2			20	
Ouaissi et al.^87^	PD+VR	59	≥1	57.6^a^	–	–	18.7	44.1
	PD	82		86.6			17.5	

SMV, superior mesenteric vein; SMA, superior mesenteric artery; PD, pancreaticoduodectomy; VR, venous resection

^a^Difference between PD+VR and PD is statistically significant (*p* < 0·05)

^b^0 mm until 2007, ≥1 mm since 2008

A French Association of Surgery study compared outcomes between patients undergoing pancreaticoduodenectomy with or without VRR and observed a significantly reduced median survival (21 vs 29 months; *p* = 0.0002) after VRR, possibly related to more advanced and aggressive disease.^35^ Interestingly, in this group, NAT administration tended to improve the prognosis. Pancreatectomy with VRR also is frequently associated with R1 resection, which can be explained by the proximity of the SMA in uncinate process cancer resulting in positive SMA margins even after VRR.^3^^,^^64^^,^^66^ Only a few studies have detailed the status of SMV/PV and/or SMA margins more specifically, with various results regarding the positivity of each margin, but with no evaluation of their survival impact.^62^^,^^82^^,^^85^ Routine VRR was proposed by Turrini et al.^88^, who matched patients undergoing pancreatectomy and VRR without vascular invasion at final pathology with patients undergoing standard pancreatectomy and observed better survival after VRR (median survival, 42 vs 22 months; *p* = 0.02). Therefore, VRR likely enlarges the SMV/PV margin and increases the chance of complete resection. Given the potential higher risk of complications and the absence of survival advantage currently demonstrated after pancreaticoduodectomy with VRR, patient candidates for this procedure should be selected according to their operative risk based on their age and comorbidities.

The pathologic invasion of the venous wall is a tumor aggressiveness marker and a predictor of worse survival.^34^^,^^37^^,^^56^^,^^62^^,^^63^^,^^89^ The impact from SMV/PV depth of invasion is not clear, but extension into the intima, the lumen, or both (compared with only the adventitia and media) may be associated with poor survival.^74^^,^^89^^,^^90^ However, the depth of venous invasion is not routinely evaluated by pathologists, and its prognostic impact is not well studied.

The presence of cancer cells at the cutting edge of the vein is another poorly evaluated feature. When the surgeon performs VRR, the exact place to cut is not easy to determine macroscopically because benign adherences can be confused with tumoral perivascular tissue. Prakash et al.^89^ evaluated the impact of having malignant cells on the venous edge without observing the survival impact of a positive venous edge and concluded that surgeons should not fear to reduce the length of VRR as long as the transection is performed through a macroscopically normal vein.

## Surgical Techniques of Vascular Reconstruction

During the last decade, notable progress has been made in surgical techniques and procedures to reduce postoperative complications and improve R0 resection rates. As recommended in the NCCN guidelines (2017), an oncologic resection needs to be performed with a meticulous perivascular dissection by skeletization of superior mesenteric vessels. The SMV/PV needs to be separated completely from the uncinate process, and in case of vein infiltration, an aggressive approach to VRR is suggested, although this concept is not universally accepted.^18^ As suggested previously, pancreatectomy and VRR should be performed in monobloc to avoid tumor-splitting in case of true vascular involvement and to locate the tumor–vessel interface precisely on the specimen.

Venous resection is either segmental or tangential, and the reconstruction technique depends on its type, its length, and the surgeon’s habit. Two studies compared the postoperative outcome according to the type of VRR, either segmental or tangential, observing similar morbidity and mortality rates.^91^^,^^92^ After segmental resection, an end-to-end anastomosis is required by direct suture or by interposition of a graft (autologous, homologous, or prosthetic). A direct end-to-end suture without a graft is limited by the length of the venous defect, which should be shorter than 3 cm to prevent tension on the suture from impaired venous patency.^93^ A tangential or “lateral” resection is reconstructed by either direct suture or patch venoplasty. The advantage of the lateral patch is that it can be sutured onto a longer venous defect required for R0 margin obtention.

With regard to using an autologous patch, the parietal peritoneum is an excellent substitute in pancreatic surgery. Dokmak et al.^94^ described the use of the peritoneal patch for SMV/PV reconstruction and observed 100% patency with the use of a lateral peritoneal patch after a mean follow-up period longer than 1 year. The peritoneal patch has other advantages including the rapidity and simplicity of harvesting through the same surgical incision and the same surgical field, particularly for emergent or unplanned situations; its inexpensive cost; the absence of increased septic risk compared with prosthetic grafts; and its good long-term venous patency. Whatever venous reconstruction is used, the portal-clamping time should be minimized and ideally limited to 30 min to avoid segmental portal hypertension, intestinal ischemia, coagulation disorders, and biologic and hemodynamical disturbances.

For tumors located in the inferior part of the uncinate process, involvement of the first SMV branches may be observed, which are considered unresectable according to NCCN guidelines.^18^ Jejuno-ileal venous branches have a small caliber with a thin and fragile wall not well suitable for reconstruction.^95^ One or two jejuno-ileal branches can be ligated in case of persistent venous flow through a residual branch and mesenteric collaterals or through reconstruction with an interposition graft, but such a procedure remains technically challenging and poorly described.^96^

## When Should the Vein be Resected?

Pancreatic cancers of the head or uncinate process often are in contact with the superior mesenteric vessels, even invading them, and are exposed to a higher risk of incomplete resection due to positive SMA and/or SMV/PV margins. To date, no reliable method exists for pre- or intraoperative differentiation between tumor infiltration of the SMV/PV and tumor-related inflammatory adherences or fibrosis after NAT. Although NAT may help to clear the tumor–vessel interface, the tumoral response to these therapies remains heterogeneous and is recognized only at the final pathologic examination.

To improve the chances of R0 resection in the absence of likelihood that the vein is not involved by the tumor, pancreatectomy with VRR can be performed safely with similar postoperative morbidity and mortality rates in the hands of an expert surgeon. The NCCN guidelines support an approach to vein resection if tumor infiltration is suspected without any additional remarks.^18^ In cases of SMV/PV with more than 180° of tumoral interface, venous irregularity, and/or short venous segment occlusion, the vein is highly susceptible to harboring malignancy and should be resected and reconstructed.^20^^,^^21^

In case of PDACs that share an interface with the SMV/PV of less than 180° without venous deformation, the probability of venous wall involvement is lower but not nil.^21^ Overall, the risk for having less than 1 mm of free margin is high, which findings have shown to be a significant prognostic factor of poor survival.^10^^,^^51^ Therefore, in the absence of reliable validated criteria, a concomitant VRR should be considered in all cases of tumoral contact with the vein (<180° and ≥180°) if the pancreatic tumor cannot be easily separated from the SMV/PV instead of continuing attempts to detach it, a process that could result in either inadvertent venous injury or a positive SMV/PV margin. This highlights the need for an accurate preoperative radiologic assessment with experienced radiologists and good-quality images.

In the future, the role of novel imaging techniques should be further evaluated, including ultrasonography for analysis of the tumor–vessel interface, especially after NAT, either preoperatively (by endoscopy) or intraoperatively. Imaging techniques such as MRI with specific sequences in combination with metabolic imaging and multisliced CT to predict the degree of tumor response to NAT together with correlative evaluation of pathologic margins and response (e.g., CAP) may be very helpful and could assist in answering the question of when the vein should be resected.^24^^,^^25^ The survival impact of VRR during pancreatectomy for tumors with SMV/PV contact is unknown, and the only current recommendation is to perform an R0 resection (with 1 mm of clearance becoming more and more accepted). Future randomized controlled trials comparing the survival outcomes for patients undergoing VRR or not in cases of PDAC surrounding mesenteric vessels (resectable and borderline) are highly desirable.
